# A validation study demonstrating portable motion capture cameras accurately characterize gait metrics when compared to a pressure-sensitive walkway

**DOI:** 10.1038/s41598-024-68402-x

**Published:** 2024-07-29

**Authors:** Kevin A. Mazurek, Leland Barnard, Hugo Botha, Teresa Christianson, Jonathan Graff-Radford, Ronald Petersen, Prashanthi Vemuri, B. Gwen Windham, David T. Jones, Farwa Ali

**Affiliations:** 1 Department of Neurology, Mayo Clinic, Rochester, MN USA; 2 Department of Radiology, Mayo Clinic, Rochester, MN USA; 3 Department of Quantitative Health Sciences, Mayo Clinic, Rochester, MN USA; 4 Department of Medicine, The MIND Center, University of Mississippi Medical Center, Jackson, MS USA

**Keywords:** Depth video, Gait analysis, Motion capture, Portable cameras, Point-cloud, Biomedical engineering, Motor control, Movement disorders, Ageing

## Abstract

Digital quantification of gait can be used to measure aging- and disease-related decline in mobility. Gait performance also predicts prognosis, disease progression, and response to therapies. Most gait analysis systems require large amounts of space, resources, and expertise to implement and are not widely accessible. Thus, there is a need for a portable system that accurately characterizes gait. Here, depth video from two portable cameras accurately reconstructed gait metrics comparable to those reported by a pressure-sensitive walkway. 392 research participants walked across a four-meter pressure-sensitive walkway while depth video was recorded. Gait speed, cadence, and step and stride durations and lengths strongly correlated (r > 0.9) between modalities, with root-mean-squared-errors (RMSE) of 0.04 m/s, 2.3 steps/min, 0.03 s, and 0.05–0.08 m for speed, cadence, step/stride duration, and step/stride length, respectively. Step, stance, and double support durations (gait cycle percentage) significantly correlated (r > 0.6) between modalities, with 5% RMSE for step and stance and 10% RMSE for double support. In an exploratory analysis, gait speed from both modalities significantly related to healthy, mild, moderate, or severe categorizations of Charleson Comorbidity Indices (ANOVA, Tukey’s HSD, *p* < 0.0125). These findings demonstrate the viability of using depth video to expand access to quantitative gait assessments.

## Introduction

The US population is aging rapidly, and soon the number of adults aged 65 and older is anticipated to outnumber children for the first time in US history^1^. With an aging population, the incidence of cognitive disorders and neurologic diseases is also anticipated to increase^2–4^. Neurological and physiological changes related to aging have numerous effects on one’s health, including decline in mobility^5^. Gait changes are a significant concern for older adults because they lead to falls, disability, and loss of independence^6,7^. Gait abnormalities can also be critical for detecting and tracking the progression of neurologic diseases^8^. Abnormalities in gait have been correlated with a higher future risk of developing dementia^9,10^. Gait speed has been shown to have a strong correlation with survival amongst older adults, where a 0.1 m/s change has been associated with increased mortality^11^. Thus, quantified gait assessments are a critical first step for identifying potential gait abnormalities and subsequently their implications on an individual’s health span.

There are numerous approaches for quantifying gait patterns using devices such as marker-based motion capture systems, walkways, treadmills, and wearables^12–16^. Instrumented walkways provide detailed spatiotemporal information about the timing of each foot fall, the step and stride lengths, asymmetry, or variability between strides, as well as numerous other gait metrics^17–19^. Camera-based motion capture systems are another established technology for characterizing the kinematics of a patient’s gait^20,21^. Traditionally, motion capture systems have involved placing reflective markers at key anatomical locations, and the marker trajectory is measured while performing different movements to derive gait features. These systems allow for accurately reconstructing the kinematic time-series which can provide crucial information for identifying abnormalities in gait using approaches such as quantitative gait analysis and other machine learning approaches^22–26^. These systems can accurately quantify changes in gait, however, they require a large, dedicated space, resources, and technical expertise to implement efficiently.

With advancements in artificial intelligence (AI) pose estimation models and gait pattern analyses^22,27^, marker-less systems using portable cameras might be able to provide information comparable to a gold standard pressure-sensitive walkway or motion capture system^28–35^. Many popular AI models are typically trained on video images available publicly (e.g., sourced from the internet) such as Google Media Pipe’s pose estimation model^36^ or OpenPose^37^. These models perform well at using color video to identify specific joint markers. The accuracy of these models has been shown to be effective for sports training, person identification, and clinical applications including gait analysis^26,38–43^. However, video based pose estimation models may include recognizable facial features in addition to gait characteristics, which increases data safety and privacy concerns for some patients^44^.

A possible solution to mitigate these privacy concerns is to analyze gait using depth sensors currently available in numerous video cameras (often termed RGB-D cameras). These technologies include a depth sensor that can provide 3D information about people or objects in the field of view, and the resulting depth data has been proposed as another approach to estimate human pose^45^. Excluding the color video from the analysis allows for characterizing gait without collecting facial features. A person’s gait has been shown to be a personally identifiable characteristic^46^, however removing the additional collection of facial features can help to reduce how much identifying information is collected. For such a system to be clinically viable, the gait metrics extracted from the point-cloud data must be validated against a gold-standard system. Here, we demonstrate a proof-of-concept system that uses depth information from two portable cameras to extract gait metrics with accuracy comparable to a pressure-sensitive walkway. These findings could lead to a portable system that can expand access to quantitative gait assessments.

## Results

Gait assessments from 392 consecutive patients enrolled in the Mayo Clinic Study of Aging (MCSA) were analyzed as part of this study. The MCSA is a population-based cohort, patients in this study were recruited from Olmsted County Minnesota using a consecutive sampling approach. The median age of the participants at the time of the walking assessment was 73 years (51 and 89 years for the 5th and 95th percentiles, respectively), with equal numbers of male and female participants (196 each). Patients walked across a pressure-sensitive walkway and were simultaneously recorded using depth video (see Methods). Gait metrics were derived from both depth video data and the walkway. These metrics are referred to as “depth video” and “walkway” metrics, respectively.

**Figure 1:**
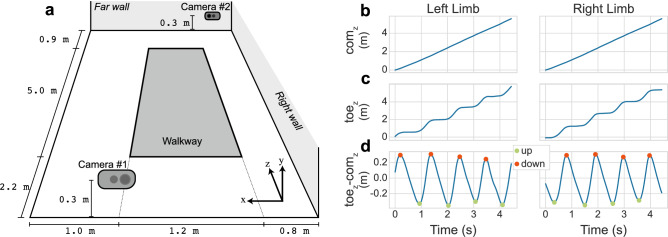
Schematic of the camera and walkway layout and the step detection algorithm for the left and right limbs (left and right panels, respectively). (a) Cartoon depiction of the placement of the walkway and cameras in the room. Participants entered the room and walked along the z-axis dimension across the walkway. The x-axis pointed to the left wall and the y-axis pointed up towards the ceiling. Camera #1 was mounted on the *near wall* and camera #2 was mounted on the *far wall* based on where physical mounts could be placed in the room. The width of the walkway was 1.2 m across, and dashed lines extend to the near wall to show how this is represented in the three-dimensional perspective of the schematic. The *far wall* and *right wall* are labeled in *italics* in the schematic, the *near wall* and *left wall* were not for simplicity of the drawing. (**b**) Position of the center of mass (com) of the body moving across the walkway in the z-direction. (**c**) Position of the toe marker moving across the walkway in the z-direction. (**d**) Difference between the toe marker and the com marker in the z-direction. Orange circles indicate peaks when the toe is maximally in front of the center of mass. Green circles indicate when the toe is maximally behind the center of mass.

**Figure 2:**
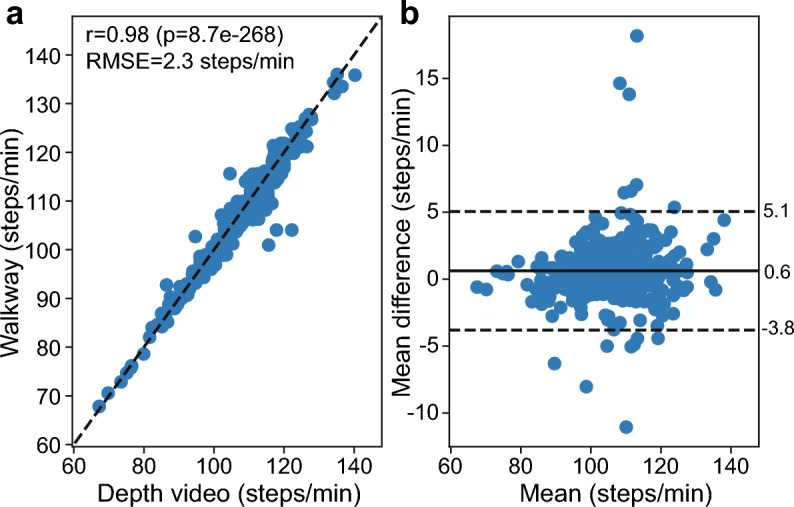
Correlation plot and Bland–Altman plot comparing cadence metrics calculated from the depth video and reported by the walkway. (**a**) Correlation plot comparing cadence for the walkway (y-axis) vs the depth video (x-axis). Black dashed line indicates y = x. Correlation, *p* value, and RMSE annotated in the plot. (**b**) Bland–Altman plot indicating the difference between the depth video and the walkway vs the average value of the two modalities. Black dashed lines indicate the lower and upper LOAs, solid line indicates mean difference. Values are depicted to the right of the plot.

Figure 1 depicts a schematic of the room in which participants walked across the walkway, as well as the center of mass (com) and toe markers for the left and right limbs for one participant. The z-direction was re-oriented to align with the forward movement of the person. Steps were identified based on the valid sequence of up and down events for the left and right limbs (see Methods for more details). The median number of strides detected by the walkway was 15 (12, 22 strides for the 5th and 95th percentiles, respectively) compared with 12 (8, 21 strides) from the depth video. Figure 2 depicts the cadence measured from the depth video and the walkway. There was a strong correlation between the two modalities (r = 0.98) with a root-mean-square error (RMSE) of 2.3 steps/min between modalities. The Bland–Altman plot^47^ shows there was a bias of + 0.6 steps/min (− 3.8, 5.1 steps/min, lower and upper limits, respectively), with 96.2% of the data within the limits of agreement (LOA), consistent with what would be expected^48^.

### Accuracy of the distance and temporal gait metrics calculated from depth information

**Figure 3:**
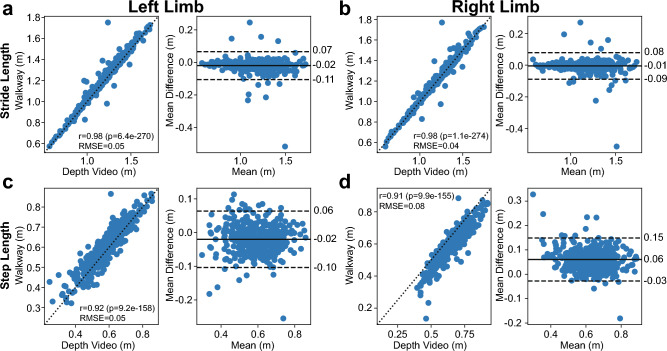
Correlation and Bland–Altman plots of distance gait metrics comparing both the left and right limbs. (**a**) Left stride length, (**b**) right stride length, (**c**) left step length, and (**d**) right step length. For each metric, the left-most plot depicts the Pearson correlation (r), *p* value, and RMSE value, and the right-most plot depicts the Bland–Altman plot with mean difference and LOAs. Conventions similar to Fig. 2.

Temporal- and distance-based measures of gait have been shown to be strong indicators of aging in older adults^11^. Here, we found strong correlations for distance and temporal gait metrics calculated from the depth video and the walkway (Table 1, Fig. 3). Left and right limb stride length metrics had correlation coefficients of 0.98 for each limb with RMSE of 0.05 m for left strides and 0.04 m for right strides. Based on the Bland–Altman plots in Fig. 3a, there was a mean difference of − 0.019 m (− 0.11, 0.07 m lower and upper LOAs, 97.2% of data within LOAs) between the depth video and the walkway for left stride lengths (negative value indicates the stride length derived from the depth video was less than the walkway measurement). For the right limb (Fig. 3b), the mean difference was − 0.005 m (− 0.09, 0.08 m lower and upper LOAs, 96.7% of data within LOAs).

**Table 1:** Precision, accuracy, and correlation of temporal and distance metrics.

Feature	Precision (RMSE)	Accuracy (mean difference)	Correlation (Pearson r)
Gait Speed (m/s)-Left	0.04	− 0.003	0.98
Gait Speed (m/s)-Right		− 0.001	
Cadence (steps/min)	2.3	0.6	0.98
Stride Length (m)-Left	0.05	− 0.01	0.98
Stride Length (m)-Right	0.04	0.06	
Stride Duration (s)-Left	0.03	− 0.003	0.98
Stride Duration (s)-Right		-0.005	
Step Length (m)-Left	0.05	− 0.02	0.92
Step Length (m)-Right	0.08	0.06	0.91
Step Duration (s)-Left	0.03	− 0.012	0.90
Step Duration (s)-Right		0.009	0.92

For the step length metrics (Fig. 3c and 3d), the left limb had a correlation of 0.92 and a RMSE of 0.05 m, and the right limb had a correlation of 0.91 and a RMSE of 0.08 m between the depth video and walkway. Bland–Altman plots again revealed a mean difference of − 0.02 m (0.06 m, − 0.10 m lower and upper LOAs, 94.9% of data within LOAs) for the left limb and 0.06 m (− 0.03, 0.15 m lower and upper LOAs, 96.7% of data within LOAs) for the right limb.

When analyzing the durations of the stride and steps, a similarly strong correlation and consistency was observed between the depth video and walkway. For the stride durations, both the left and right limb metrics had correlations of 0.98 and RMSEs of 0.03 s for each limb (Fig. 3c). The left limb stride duration had a mean difference of − 0.003 s (− 0.05, 0.05 s lower and upper LOAs, 95.9% of data within LOAs). Right limb stride durations had a mean bias of − 0.005 s (− 0.05, 0.04 s lower and upper LOAs, 95.7% of data within LOAs). Similarly for the step durations (Fig. 3d), the left limb had a correlation of 0.90 and the right limb had a correlation of 0.92, with 0.03 s RMSE for each limb. The Bland–Altman plots indicated there was a − 0.012 s mean difference (− 0.07, 0.04 s, lower and upper LOAs, 93.9% of data within LOAs) for the left limb and a + 0.009 s bias (− 0.09, 0.08 s, lower and upper LOAs, 93.4% of data within LOAs) for the right limb.

Measures of gait speed, calculated by dividing the stride length by the stride duration, also showed strong correlations between the depth video and the walkway (r = 0.98 both left and right limbs) with RMSEs of 0.04 m/s for each limb. The Bland–Altman analyses revealed a mean difference of − 0.003 m/s (− 0.09, 0.08 m/s lower and upper LOAs, 96.7% of data within LOAs) for the gait speed calculated from left strides and − 0.001 m/s (− 0.09, 0.08 m/s lower and upper LOAs, 97.2% of data with LOAs) for the gait speed calculated from right strides.

### Accuracy of identifying different gait cycle phases depth information

**Figure 4:**
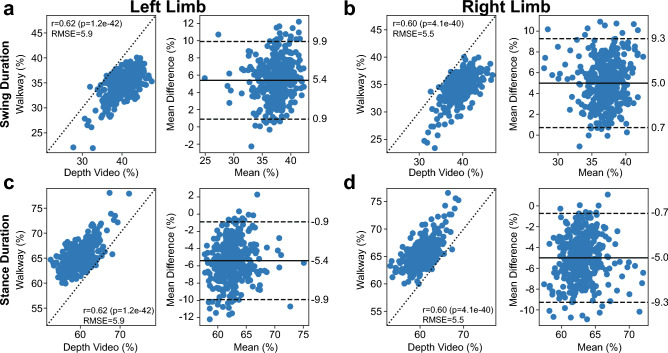
Correlations and Bland–Altman plots for the phases of the gait cycle. (**a**) Left limb swing duration, (**b**) Right Limb swing duration, (**c**) Left limb stance duration, and d) Right limb stance duration. Each metric is represented as a percentage of the gait cycle. For each metric, the left-most plot depicts the Pearson correlation (r), *p* value, and RMSE value, and the right-most plot depicts the Bland–Altman plot with mean difference and LOAs. Conventions similar to Fig. 2.

Next, we examined how well we could determine the duration of different phases of the step cycle (compared to those detected by the pressure-sensitive walkway). Swing duration, stance duration, and double support duration were each reported as a percentage of gait cycle (Table 2). When comparing the depth video and walkway metrics (Fig. 4a and 4b), the swing duration had a correlation of 0.62 for the left limb and 0.60 for the right limb. The left limb swing duration had a 6% RMSE and the right limb swing duration had a 5% RMSE. Bland–Altman plots revealed that both metrics had a mean difference of 5 percentage points (0.9, 9.9% lower and upper LOAs for left, 92.9% of data within LOAs; 0.7, 9.3% lower and upper LOAs for right, 95.9% of data within LOAs). For the stance duration (Fig. 4c and 4d), the correlations were again 0.62 for the left limb and 0.60 for the right limb, and 6% and 5% RMSE for the left and right limb stance duration, respectively. Based on the Bland–Altman plots, there was a mean difference of − 5 percentage points for both metrics (− 9.9, − 0.9% lower and upper LOAs for left, 92.9% of data within LOAs, − 9.3, − 0.7 lower and upper LOAs for right, 95.9% of data within LOAs). For the double stance duration, the correlation was 0.69, 11% RMSE, and mean difference of − 10 percentage points for each limb (− 17.2, − 2.9% lower and upper LOAs for left, 94.9% of data within LOAs; − 17.4, − 3.2% lower and upper LOAs, 95.2% of data within LOAs). Overall, the symmetry with which the depth video overestimated the stance duration and underestimated the swing phase could be due to differences with how the toe down and toe up events were detected between modalities.

**Table 2:** Precision, Accuracy, and Correlation of Gait Cycle Phases.

Feature	Precision (RMSE)	Accuracy (mean difference)	Correlation (Pearson r)
Swing (%GC)-Left	6	5	0.62
Swing (%GC)-Right	5		0.60
Stance (%GC)-Left	6	− 5	0.62
Stance (%GC)-Right	5		0.60
Double support (%GC)-Left	11	− 10	0.62
Double support (%GC)-Right			0.60

### Gait Speed significantly relates to comorbidity scores similarly for both modalities

**Figure 5:**
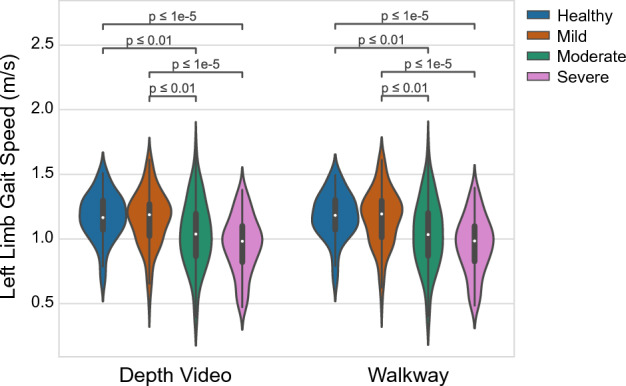
Comparison of left limb gait speed with discretized CCI scores. CCI scores are categorized into Healthy (blue), Mild (dark orange), Moderate (green), and Severe (light purple). Significance bars are displayed at the top with p-values after performing Tukey’s HSD post-hoc tests between groups. Metrics derived from the depth videos shown on the left, reported by the walkway shown on the right.

Several studies have identified that reduced gait speed is a significant predictor of increased risk of mortality in older adults^11^. As an exploratory analysis, we selected a subset of 336 participants for which we had Charleson Comorbidity Index (CCI) scores^49^. Participants were categorized into healthy, mild, moderate, or severe morbidity risk if their CCI scores were 0, 1–2, 3–4, and ≥ 5, respectively. Of the 336 participants, 58 were healthy (17.26%), 100 were mild (29.76%), 73 were moderate (21.73%), and 105 were severe (31.25%). There was a significant effect of gait speed for the different CCI categories (Fig. 5, ANOVA, *p* < 0.025). Based on the Tukey’s HSD post-hoc tests, the gait velocity for individuals categorized as healthy or mild was greater than those categorized as moderate (*p* < 0.01) or severe (*p* < 1e−5) individuals. However, there was no significant difference between healthy and mild, or moderate and severe. The same pattern was detected from gait velocities for the right limb, with the same statistical values (ANOVA, *p* < 0.025, Tukey’s HSD *p* < 0.01 for healthy or mild vs moderate and *p* < 1e−5 for healthy or mild vs. severe). Importantly, the outcomes were identical using either the depth video or walkway to derive the gait metrics, demonstrating depth video can extract gait metrics with clinically meaningful information.

## Discussion

We have successfully demonstrated that depth information collected from two portable cameras can accurately reconstruct gait metrics on par with a gold standard pressure-sensitive walkway. There was a strong correlation between measurements made by the two modalities for distance measures (e.g., step length and step duration) which are important clinical markers for assessing gait abnormalities. High degree of agreement with small errors demonstrated validity while small RMSE values demonstrated precision of the depth video compared to the pressure-sensitive walkway. The agreement between the different phases of the gait cycle was fairly accurate but not as strong as the distance measures. This could be due to differences in when the walkway detects the foot lifting and pressing down on the ground compared to what was derived from the depth video. The depth video detected these events based on when the toe marker was maximally in front or behind the body center-of-mass. The toe marker may begin to make contact or release from the walkway before these points, which could explain the difference. However, the kinematic video information does not have the same kinetic precision as the walkway and thus a more refined foot detection algorithm could resolve these differences (e.g., by using machine learning approaches to identify the timing of toe down and toe up against the two modalities). Importantly, the depth video metrics were consistent with the walkway for each of the gait metrics calculated. The statistical pattern relating gait velocity to CCI scores (Fig. 5) demonstrates that the metrics derived are similar between modalities but also relate to clinically meaningful measures. Overall, these findings demonstrate the utility of such a system for clinical use. The population assessed in this study was relatively normal in relation to known movement disorders, so future work would be needed to validate the technology on patients with more pronounced gait abnormalities such as patients with Parkinson’s disease or normal pressure hydrocephalus. However, the findings here are a promising step toward remotely tracking aging or neurologic disease progression using gait metrics^8^.

The proposed system only uses depth (point-cloud) data to track the joint positions. This is advantageous from a patient privacy perspective because although gait is an identifying characteristic, omitting the use of recognizable facial and body features at least removes further identifying information to alleviate some privacy concerns^44^. Depth cameras have been shown to be limited in their capture rate and the size of the capture space which can affect the accuracy of detecting individual joint positions during walking^50,51^. Incorporating color video to supplement the point-cloud data and potentially improve marker tracking could be helpful, however at the tradeoff of requiring more identifying features (e.g., facial features) to be recorded. Another potential limitation of the proposed setup is human intervention is required to process the depth video and track the skeletal markers. Although a fully automated system would be more efficient, this human intervention step provides a quality control check to ensure the tracking software accurately detected the person in view. Additionally, in our study the tracking step was brief to perform given the nature of the movements being tracked.

Overall, the agreement of these systems suggests a portable system could be a viable solution for performing initial gait assessments for patients at medical centers where implementing a more resource intensive gait lab might not be possible. This approach should not be considered as a replacement for pressure-sensitive walkways or motion capture systems. The portability of this system also expands access for performing longitudinal assessments of gait which is crucial for early detection of potential gait abnormalities and tracking gait-based biomarkers related to the progression of different neurologic diseases. In this cohort of relatively healthy individuals, minor differences were observed between the gait metrics reported by the pressure-sensitive walkway and the depth video, such as differences in footfall detection or offsets swing and stance phase durations. However, these differences were within acceptable limits when assessing the accuracy and precision of gait metrics between modalities. Before implementing such a system for clinical applications, further research is needed to assess the system’s accuracy and precision in a diverse population of patients with different neurologic diseases or movement disorders to ensure the performance is within acceptable limits. Clinical use would require further validation and assessment of accuracy and precision in light of clinically meaningful change and disease heterogeneity.

## Methods

### Participants

We conducted a validation study of 392 participants as part of the Mayo Clinic Study of Aging (MCSA) using a consecutive sampling approach. Part of their assessments involves walking across a 5 m pressure-sensitive walkway (ZenoTM Walkway, ProtoKinetics LLC, Havertown, Pennsylvania). Participants consented to also have depth information be recorded as part of this study. All participants provided informed consent prior to their inclusion in the study, and all procedures and protocols of this study conformed to the Declaration of Helsinki and were approved by the Mayo Clinic Institutional Review Board.

### Walking assessment

Enrolled participants performed a walking assessment in which they walked over a 5 m pressure-sensitive walkway two times at their own pace. A schematic of the room is depicted in Fig. 1a. Study coordinators instructed the participants to stand at the entrance of the assessment room, walk straight across the assessment room, turn around, and walk back to their starting point at their normal speed. Participants began walking and turned around while not on the walkway such that the averaged gait metrics from each step more closely corresponded to their steady state walking. Two Azure HD Kinect (Microsoft, Washington, USA) cameras were placed in the room which were synchronized to a laptop using the iPi Recorder software (iPi Soft LLC, Lamarjean Group, Inc, Poway, CA) to record depth information related to their walking. The cameras nominally recorded at 30 frames per second (FPS), however for some sessions the frame rate dropped due to issues synchronizing the two cameras. For these sessions, we used the average frame rate reported by the iPi Recorder when analyzing the data. Although the cameras can record both RGB and depth information, only depth information was recorded here to prevent recording personally identifiable information such as facial features. Both cameras were calibrated using a small flashlight as described by the manufacturer’s instructions. In short, this involved moving a small flashlight throughout the capture space while recording a calibration file using the iPi Recorder software. This calibration file was then used for analyzing the subsequent patient recordings. The positions of the cameras were placed based on the physical configurations of the room as well as the manufacturer’s recommendations for the capture space dimensions. As depicted in Fig. 1a, the walkway was 0.9 m from the far wall and 2.2 m from the near wall. Two Azure HD Kinect cameras were mounted 0.3 m above the ground on the near wall (camera #1) and the far wall (camera #2). Based on physical restrictions in the room, the walkway was not completely centered, with the walkway being 1.0 m from the left wall and 0.8 m from the right wall.

### Depth video data preprocessing and extracting gait metrics

The depth recordings were processed using the iPi Mocap Studio 4 Software (iPi Soft LLC). A skeleton was fit to the depth point cloud data to track the person walking during the assessment. The turn was excluded from analyses because only one camera had coverage which was insufficient for accurately tracking. The skeleton trajectory was smoothed using the iPiSoft jitter removal tool to remove potential outlier datapoints. Once the depth data were processed, the joint coordinates were exported using the biomech add-in to obtain absolute positions relative to the global ground in the tracking software. The default skeleton available in the iPi Motion Studio provided the following marker coordinates: center of mass, center of mass projection to ground, hip, lower spine, middle spine, chest, neck, head, eye, effector head, clavicle (Left/Right), shoulder (L/R), forearm (L/R), hand (L/R), thigh (L/R), shin (L/R), foot (L/R), toe (L/R), effector toe (L/R). The cartesian values of the joint coordinates were outputted in tabular format text files which were processed in python for subsequent analysis. These text files contained header information to select specific joint marker information, such as $toe$ or $com$ coordinates, for subsequent stride detection. Placement of the cameras did not capture when participants began walking or when they turned around to be consistent with the gait information collected by the walkway.

The center of mass ($com$) and left and right toe markers were used for detecting strides during the session. Any missing frames were interpolated using piecewise cubic hermite interpolating polynomial (pchip) algorithm^52^. Next, we confirmed the person was walking straight and not turning in the middle of the session. We used the $co{m}_{x}$ and $co{m}_{z}$ positions and subtracted the first frame to ensure the person was walking forward in the $co{m}_{z}$ direction. Turns were identified based on positive or negative $co{m}_{z}$ peaks (scipy find_peaks function, prominence = 1). Time steps between these turn events were excluded from additional analysis. The $co{m}_{z}$ and $to{e}_{z}$ positions were used to detect strides. Strides were defined from toe-down to toe-down (${t}_{down\left[n+1\right]}-{t}_{down\left[n\right]}$) of the ipsilateral limb. First, the $to{e}_{z}$ position was subtracted by the $co{m}_{z}$ position to find the relative change in the foot from the body. Toe down was defined as the positive peak of $to{e}_{z}$ from the $co{m}_{z}$ (find_peaks, prominence 0.5 standard deviations of the overall $to{e}_{z}$). Similarly, toe up was defined as the negative peak of $to{e}_{z}$ from the $co{m}_{z}$ (in the backward direction). These events were then labeled as ${R}_{down}$, ${R}_{up}$, ${L}_{down}$, and ${L}_{up}$ depending on which $to{e}_{z}$ marker was being analyzed. This resulted in a sequence of events that was then used to detect “valid strides.” We defined a valid right stride as sequence of ${R}_{down}-{L}_{up}-{L}_{down}-{R}_{up}-{R}_{down}$ and left stride as ${L}_{down}-{R}_{up}-{R}_{down}-{L}_{up}-{L}_{down}$. These events also defined the gait cycle for which subsequent metrics were calculated.

Using the detected strides, we calculated the following gait metrics similar to those described in^53^: (1) Stride duration: time between toe down events (${t}_{down\left[n+1\right]}-{t}_{down\left[n\right]}$); (2) Stride distance: Euclidean distance between toe down events, $\left|{toe}_{x,z}\left({t}_{down\left[n+1\right]}\right)-{toe}_{x,z}\left({t}_{down\left[n\right]}\right)\right|$; (3) step duration: time from the ipsilateral toe down to the contralateral toe down (${t}_{contr,down\left[n\right]}-{t}_{ips,down\left[n\right]}$); (4) step distance: Euclidean distance between the ipsilateral and contralateral toe down events $\left|{contr,toe}_{x,z}\left({t}_{down\left[n+1\right]}\right)-{ips, toe}_{x,z}\left({t}_{down\left[n\right]}\right)\right|$; the following time based metrics were represented as a percentage of the gait cycle (5) double support: duration both feet were on the ground $100\times \left(\left({t}_{contr,up\left[n\right]}-{t}_{ips,down\left[n\right]}\right)+\left({t}_{ips,up\left[n\right]}-{t}_{contr,down\left[n\right]}\right)\right)/\left({t}_{ips,down\left[n+1\right]}-{t}_{ips,down\left[n\right]}\right)$; (6) swing duration: time from ipsilateral toe lifts up to touches down $\left({t}_{ips,up\left[n\right]}-{t}_{ips,down\left[n+1\right]}\right)/\left({t}_{ips,down\left[n+1\right]}-{t}_{ips,down\left[n\right]}\right)$; (7) stance duration: time from the contralateral toe lifts up to touches down $\left({t}_{contr,up\left[n\right]}-{t}_{contr,down\left[n+1\right]}\right)/\left({t}_{ips,down\left[n+1\right]}-{t}_{ips,down\left[n\right]}\right)$; (8) gait speed: stride length divided by stride time; and (9) cadence: the sum of the strides for both limbs divided by the sum of the stride durations calculated for the entire session.

### Exploratory analysis comparing Charleson Comorbidity Index (CCI) to gait speed

The Charleson Comorbidity Index (CCI) scores provide a morbidity score reflecting one’s mortality risk^49^. To calculate the score, 19 different medical conditions are assessed in addition to including a risk factor for the age of the patient. For a subset of 336 patients, CCI scores were available which were then divided into 4 categories similar to those reported by Huang et al.^54^: healthy/no-comorbidity (CCI = 0), mild (CCI = 1 or 2), moderate (CCI = 3 or 4), severe (CCI ≥ 5). As an exploratory analysis, we compared the gait speed metric from the left and right limbs separately to determine how changes in gait speed related to increased risk of mortality based on the CCI categories. Additionally, the distributions were qualitatively compared when using gait speed derived from the depth video and reported by the walkway to examine the efficacy of using depth video metrics for clinical assessments. The seaborn python package^55^ was used to create the violin plots comparing groups, and the statsmodels annotations package^56^ was used to add significance bars for the post-hoc comparisons.

### Statistical analyses

Each of the gait metrics was compared between the depth video and pressure to those reported by the pressure-sensitive walkway. Pearson correlations and Bland–Altman plots were calculated to determine how well the values aligned for each metric. Bland–Altman results were presented as the bias of the difference between the two modalities (walkway, depth video) along with the lower and upper limits of agreement (LOAs, 1.96 × standard deviation)^47,57^. For the exploratory analysis, the Statsmodels python library^58^ was used to perform ANOVA and Tukey’s HSD post-hoc tests. The significance level for the post-hoc tests was Bonferroni corrected for testing both left and right limb gait metrics for both modalities (alpha = 0.05/(2 limbs × 2 modalities) = 0.0125).

## Data availability statement

The datasets generated during and/or analyzed during the current study are available from the corresponding author on reasonable request.

## References

[1] Vespa, J., Armstrong, D. M. & Medina, L. *Demographic turning points for the United States: Population projections for 2020 to 2060* (US Department of Commerce, Economics and Statistics Administration, US, 2018).

[2] Nichols, E. *et al.* Estimation of the global prevalence of dementia in 2019 and forecasted prevalence in 2050: an analysis for the Global Burden of Disease Study 2019. *Lancet Public Health* **7**, e105–e125. 10.1016/S2468-2667(21)00249-8 (2022). 34998485 10.1016/S2468-2667(21)00249-8 PMC8810394

[3] Rajan, K. B. *et al.* Population estimate of people with clinical Alzheimer’s disease and mild cognitive impairment in the United States (2020–2060). *Alzheimers Dement* **17**, 1966–1975. 10.1002/alz.12362 (2021). 34043283 10.1002/alz.12362 PMC9013315

[4] Hebert, L. E., Weuve, J., Scherr, P. A. & Evans, D. A. Alzheimer disease in the United States (2010–2050) estimated using the 2010 census. *Neurology* **80**, 1778–1783. 10.1212/WNL.0b013e31828726f5 (2013). 23390181 10.1212/WNL.0b013e31828726f5 PMC3719424

[5] Briggs, A. M. *et al.* Musculoskeletal health conditions represent a global threat to healthy aging: A report for the 2015 World Health Organization world report on ageing and health. *Gerontologist* **56**(Suppl 2), S243-255. 10.1093/geront/gnw002 (2016). 26994264 10.1093/geront/gnw002

[6] Verghese, J., Ambrose, A. F., Lipton, R. B. & Wang, C. Neurological gait abnormalities and risk of falls in older adults. *J. Neurol.* **257**, 392–398. 10.1007/s00415-009-5332-y (2010). 19784714 10.1007/s00415-009-5332-y PMC2838981

[7] Quach, L. *et al.* The nonlinear relationship between gait speed and falls: The maintenance of balance, independent living, intellect, and zest in the elderly of Boston study. *J. Am. Geriatr. Soc.* **59**, 1069–1073. 10.1111/j.1532-5415.2011.03408.x (2011). 21649615 10.1111/j.1532-5415.2011.03408.x PMC3141220

[8] Celik, Y., Stuart, S., Woo, W. L. & Godfrey, A. Gait analysis in neurological populations: Progression in the use of wearables. *Med. Eng. Phys.* **87**, 9–29. 10.1016/j.medengphy.2020.11.005 (2021). 33461679 10.1016/j.medengphy.2020.11.005

[9] Verghese, J. *et al.* Abnormality of gait as a predictor of non-Alzheimer’s dementia. *N. Engl. J. Med.* **347**, 1761–1768. 10.1056/NEJMoa020441 (2002). 12456852 10.1056/NEJMoa020441

[10] Beauchet, O. *et al.* Poor gait performance and prediction of dementia: Results from a meta-analysis. *J. Am. Med. Dir. Assoc.* **17**, 482–490. 10.1016/j.jamda.2015.12.092 (2016). 26852960 10.1016/j.jamda.2015.12.092 PMC5319598

[11] Studenski, S. *et al.* Gait speed and survival in older adults. *JAMA* **305**, 50–58. 10.1001/jama.2010.1923 (2011). 21205966 10.1001/jama.2010.1923 PMC3080184

[12] Hulleck, A. A., Menoth Mohan, D., Abdallah, N., El Rich, M. & Khalaf, K. Present and future of gait assessment in clinical practice: Towards the application of novel trends and technologies. *Front. Med. Technol.* **4**, 901331. 10.3389/fmedt.2022.901331 (2022). 36590154 10.3389/fmedt.2022.901331 PMC9800936

[13] Cappozzo, A. Gait analysis methodology. *Hum. Mov. Sci.* **3**, 27–50 (1984). 10.1016/0167-9457(84)90004-6

[14] Simon, S. R. Quantification of human motion: Gait analysis-benefits and limitations to its application to clinical problems. *J. Biomech.* **37**, 1869–1880. 10.1016/j.jbiomech.2004.02.047 (2004). 15519595 10.1016/j.jbiomech.2004.02.047

[15] Topham, L. K., Khan, W., Al-Jumeily, D. & Hussain, A. Human body pose estimation for gait identification: A comprehensive survey of datasets and models. *ACM Comput. Surv.* **55**, 1–42 (2022). 10.1145/3533384

[16] Sethi, D., Bharti, S. & Prakash, C. A comprehensive survey on gait analysis: History, parameters, approaches, pose estimation, and future work. *Artif. Intell. Med.* **129**, 102314. 10.1016/j.artmed.2022.102314 (2022). 35659390 10.1016/j.artmed.2022.102314

[17] McDonough, A. L., Batavia, M., Chen, F. C., Kwon, S. & Ziai, J. The validity and reliability of the GAITRite system’s measurements: A preliminary evaluation. *Arch. Phys. Med. Rehabil.* **82**, 419–425. 10.1053/apmr.2001.19778 (2001). 11245768 10.1053/apmr.2001.19778

[18] Bilney, B., Morris, M. & Webster, K. Concurrent related validity of the GAITRite walkway system for quantification of the spatial and temporal parameters of gait. *Gait Posture* **17**, 68–74. 10.1016/s0966-6362(02)00053-x (2003). 12535728 10.1016/s0966-6362(02)00053-x

[19] Vallabhajosula, S., Humphrey, S. K., Cook, A. J. & Freund, J. E. Concurrent validity of the Zeno walkway for measuring spatiotemporal gait parameters in older adults. *J. Geriatr. Phys. Ther.* **42**, E42–E50. 10.1519/JPT.0000000000000168 (2019). 29286982 10.1519/JPT.0000000000000168

[20] Carse, B., Meadows, B., Bowers, R. & Rowe, P. Affordable clinical gait analysis: An assessment of the marker tracking accuracy of a new low-cost optical 3D motion analysis system. *Physiotherapy* **99**, 347–351 (2013). 23747027 10.1016/j.physio.2013.03.001

[21] Pfister, A., West, A. M., Bronner, S. & Noah, J. A. Comparative abilities of Microsoft Kinect and Vicon 3D motion capture for gait analysis. *J. Med. Eng. Technol.* **38**, 274–280 (2014). 24878252 10.3109/03091902.2014.909540

[22] Horst, F., Lapuschkin, S., Samek, W., Muller, K. R. & Schollhorn, W. I. Explaining the unique nature of individual gait patterns with deep learning. *Sci. Rep.* **9**, 2391. 10.1038/s41598-019-38748-8 (2019). 30787319 10.1038/s41598-019-38748-8 PMC6382912

[23] Ben Chaabane, N. *et al.* Quantitative gait analysis and prediction using artificial intelligence for patients with gait disorders. *Sci. Rep.* **13**, 23099. 10.1038/s41598-023-49883-8 (2023). 38155189 10.1038/s41598-023-49883-8 PMC10754876

[24] Moissenet, F., Leboeuf, F. & Armand, S. Lower limb sagittal gait kinematics can be predicted based on walking speed, gender, age and BMI. *Sci. Rep.* **9**, 9510. 10.1038/s41598-019-45397-4 (2019). 31267006 10.1038/s41598-019-45397-4 PMC6606631

[25] Jing, Y. *et al.* Deep learning-assisted gait parameter assessment for neurodegenerative diseases: Model development and validation. *J. Med. Internet Res.* **25**, e46427. 10.2196/46427 (2023). 37405831 10.2196/46427 PMC10357315

[26] Pradhan, C. *et al.* Automated classification of neurological disorders of gait using spatio-temporal gait parameters. *J. Electromyogr. Kinesiol.* **25**, 413–422. 10.1016/j.jelekin.2015.01.004 (2015). 25725811 10.1016/j.jelekin.2015.01.004

[27] Zhou, Y. *et al.* The detection of age groups by dynamic gait outcomes using machine learning approaches. *Sci. Rep.* **10**, 4426. 10.1038/s41598-020-61423-2 (2020). 32157168 10.1038/s41598-020-61423-2 PMC7064519

[28] Ceseracciu, E., Sawacha, Z. & Cobelli, C. Comparison of markerless and marker-based motion capture technologies through simultaneous data collection during gait: proof of concept. *PLoS ONE* **9**, e87640. 10.1371/journal.pone.0087640 (2014). 24595273 10.1371/journal.pone.0087640 PMC3942307

[29] Bertram, J. *et al.* Accuracy and repeatability of the Microsoft Azure Kinect for clinical measurement of motor function. *PLoS ONE* **18**, e0279697. 10.1371/journal.pone.0279697 (2023). 36701322 10.1371/journal.pone.0279697 PMC9879399

[30] Tsai, Z. R., Kuo, C. C., Wang, C. J., Tsai, J. J. P. & Chou, H. H. Validation of gait measurements on short-distance walkways using Azure Kinect DK in patients receiving chronic hemodialysis. *J. Pers. Med.*10.3390/jpm13071181 (2023). 37511793 10.3390/jpm13071181 PMC10381698

[31] Arizpe-Gómez, P., Harms, K., Janitzky, K., Witt, K. & Hein, A. Towards automated self-administered motor status assessment: Validation of a depth camera system for gait feature analysis. *Biomed. Signal Process. Control* **87**, 105352 (2024). 10.1016/j.bspc.2023.105352

[32] Geerse, D. J., Coolen, B. H. & Roerdink, M. Kinematic validation of a multi-Kinect v2 instrumented 10-meter walkway for quantitative gait assessments. *PLoS ONE* **10**, e0139913. 10.1371/journal.pone.0139913 (2015). 26461498 10.1371/journal.pone.0139913 PMC4603795

[33] Guess, T. M., Bliss, R., Hall, J. B. & Kiselica, A. M. Comparison of Azure Kinect overground gait spatiotemporal parameters to marker based optical motion capture. *Gait Posture* **96**, 130–136. 10.1016/j.gaitpost.2022.05.021 (2022). 35635988 10.1016/j.gaitpost.2022.05.021

[34] Albert, J. A. *et al.* Evaluation of the pose tracking performance of the Azure Kinect and Kinect v2 for gait analysis in comparison with a gold standard: A pilot study. *Sensors (Basel)*10.3390/s20185104 (2020). 32911651 10.3390/s20185104 PMC7571213

[35] Latorre, J., Colomer, C., Alcaniz, M. & Llorens, R. Gait analysis with the Kinect v2: Normative study with healthy individuals and comprehensive study of its sensitivity, validity, and reliability in individuals with stroke. *J. Neuroeng. Rehabil.* **16**, 97. 10.1186/s12984-019-0568-y (2019). 31349868 10.1186/s12984-019-0568-y PMC6660692

[36] Bazarevsky, V. & Grishchenko, I. On-device, real-time body pose tracking with mediapipe blazepose. *Google AI Blog* (2020).

[37] Cao, Z., Hidalgo, G., Simon, T., Wei, S. E. & Sheikh, Y. OpenPose: Realtime multi-person 2D pose estimation using part affinity fields. *IEEE Trans. Pattern Anal. Mach. Intell.* **43**, 172–186. 10.1109/TPAMI.2019.2929257 (2021). 31331883 10.1109/TPAMI.2019.2929257

[38] Lu, M. *et al.* Vision-based estimation of MDS-UPDRS gait scores for assessing Parkinson’s disease motor severity. *Med. Image Comput. Comput. Assist. Interv.* **12263**, 637–647. 10.1007/978-3-030-59716-0_61 (2020). 33103164 10.1007/978-3-030-59716-0_61 PMC7585545

[39] Yamamoto, M., Shimatani, K., Ishige, Y. & Takemura, H. Verification of gait analysis method fusing camera-based pose estimation and an IMU sensor in various gait conditions. *Sci. Rep.* **12**, 17719. 10.1038/s41598-022-22246-5 (2022). 36271241 10.1038/s41598-022-22246-5 PMC9586966

[40] Wade, L., Needham, L., McGuigan, P. & Bilzon, J. Applications and limitations of current markerless motion capture methods for clinical gait biomechanics. *PeerJ* **10**, e12995. 10.7717/peerj.12995 (2022). 35237469 10.7717/peerj.12995 PMC8884063

[41] Bridgeman, L., Volino, M., Guillemaut, J. -Y. & Hilton, A. Multi-person 3d pose estimation and tracking in sports. In *Proceedings of the IEEE/CVF conference on computer vision and pattern recognition workshops* (2019).

[42] Topham, L. K., Khan, W., Al-Jumeily, D., Waraich, A. & Hussain, A. J. Gait identification using limb joint movement and deep machine learning. *IEEE Access* **10**, 100113–100127 (2022). 10.1109/ACCESS.2022.3207836

[43] Yagi, K., Sugiura, Y., Hasegawa, K. & Saito, H. Gait measurement at home using a single RGB camera. *Gait Posture* **76**, 136–140 (2020). 31812791 10.1016/j.gaitpost.2019.10.006

[44] Richardson, J. P. *et al.* Patient apprehensions about the use of artificial intelligence in healthcare. *NPJ Digit. Med.* **4**, 140. 10.1038/s41746-021-00509-1 (2021). 34548621 10.1038/s41746-021-00509-1 PMC8455556

[45] Zhou, Y., Dong, H. & El Saddik, A. Learning to estimate 3d human pose from point cloud. *IEEE Sens. J.* **20**, 12334–12342 (2020). 10.1109/JSEN.2020.2999849

[46] Wang, L., Tan, T., Ning, H. & Hu, W. Silhouette analysis-based gait recognition for human identification. *IEEE Trans. Pattern Anal. Mach. Intell.* **25**, 1505–1518 (2003). 10.1109/TPAMI.2003.1251144

[47] Bland, J. M. & Altman, D. Statistical methods for assessing agreement between two methods of clinical measurement. *Lancet* **327**, 307–310 (1986). 10.1016/S0140-6736(86)90837-8 2868172

[48] Myles, P. S. & Cui, J. Using the Bland–Altman method to measure agreement with repeated measures. *Br. J. Anaesth.* **99**, 309–311. 10.1093/bja/aem214 (2007). 17702826 10.1093/bja/aem214

[49] Charlson, M. E., Pompei, P., Ales, K. L. & MacKenzie, C. R. A new method of classifying prognostic comorbidity in longitudinal studies: Development and validation. *J. Chronic Dis.* **40**, 373–383. 10.1016/0021-9681(87)90171-8 (1987). 3558716 10.1016/0021-9681(87)90171-8

[50] Clark, R. A., Mentiplay, B. F., Hough, E. & Pua, Y. H. Three-dimensional cameras and skeleton pose tracking for physical function assessment: A review of uses, validity, current developments and Kinect alternatives. *Gait Posture* **68**, 193–200 (2019). 30500731 10.1016/j.gaitpost.2018.11.029

[51] Sarbolandi, H., Lefloch, D. & Kolb, A. Kinect range sensing: Structured-light versus Time-of-Flight Kinect. *Comput. Vis. Image Underst.* **139**, 1–20 (2015). 10.1016/j.cviu.2015.05.006

[52] Fritsch, F. N. & Butland, J. A method for constructing local monotone piecewise cubic interpolants. *SIAM J. Sci. Stat. Comput.* **5**, 300–304 (1984). 10.1137/0905021

[53] Huxham, F., Gong, J., Baker, R., Morris, M. & Iansek, R. Defining spatial parameters for non-linear walking. *Gait Posture* **23**, 159–163. 10.1016/j.gaitpost.2005.01.001 (2006). 16399511 10.1016/j.gaitpost.2005.01.001

[54] Huang, Y. Q. *et al.* Charlson comorbidity index helps predict the risk of mortality for patients with type 2 diabetic nephropathy. *J. Zhejiang Univ. Sci. B* **15**, 58–66. 10.1631/jzus.B1300109 (2014). 24390745 10.1631/jzus.B1300109 PMC3891119

[55] Waskom, M. L. Seaborn: Statistical data visualization. *J. Open Source Softw.* **6**, 3021 (2021). 10.21105/joss.03021

[56] Statsannotations v. v0.6 (Zenodo, 2022).

[57] Bunce, C. Correlation, agreement, and Bland–Altman analysis: Statistical analysis of method comparison studies. *Am. J. Ophthalmol.* **148**, 4–6. 10.1016/j.ajo.2008.09.032 (2009). 19540984 10.1016/j.ajo.2008.09.032

[58] Seabold, S. & Perktold, J. In *Proceedings of the 9th Python in Science Conference* 10–25080 (Austin, TX).

